# The prevalence of HBsAg, knowledge and practice of hepatitis B prevention among pregnant women in the Limbe and Muyuka Health Districts of the South West region of Cameroon: a three-year retrospective study

**DOI:** 10.11604/pamj.2019.32.122.16055

**Published:** 2019-03-15

**Authors:** Esum Mathias Eyong, Brenda Mbouamba Yankam, Esemu Seraphine, Che Henry Ngwa, Ngwayu Claude Nkfusai, Cho Sebastine Anye, Gilbert Karngong Nfor, Samuel Nambile Cumber

**Affiliations:** 1 Department of Microbiology and Parasitology, Faculty of Science, University of Buea, Buea, Cameroon; 2 Department of Statistics, Faculty of Physical Science, University of Nsukka, Nsukka, Nigeria; 3 Cameroon Baptist Convention Health Services (CBCHS), Yaounde, Cameroon; 4 Section for Epidemiology and Social Medicine, Department of Public Health and Community Medicine, Institute of Medicine (EPSO), the Sahlgrenska Academy at University of Gothenburg, Gothenburg, Sweden; 5 Faculty of Health Sciences, University of the Free State, Bloemfontein, South Africa

**Keywords:** Hepatitis B virus, prevalence, pregnant women, knowledge, practice

## Abstract

**Introduction:**

hepatitis B infection is caused by the hepatitis B virus (HBV). HBV is transmitted through sexual intercourse, by exchange of saliva during kissing and also to newborns of infected mothers. In the Global Burden of Diseases 2010, 786,000 deaths were attributed to HBV. Studies in Cameroon, reported the prevalence of HBV as high as 10.1% and 12% among blood donors in hospital blood banks. This study therefore, aims at determining the prevalence of HBsAg, knowledge and practices of pregnant women on HBV prevention and transmission in the Limbe Health District (LHD) and Muyuka Health District (MHD).

**Methods:**

ANC registers were exploited from the health centers for a period of three years (2014-2016) in order to determine the prevalence of HBV infection. 270 women attending ANC were selected by exhaustive sampling. Knowledge and practices of participants on HBV prevention and transmission was assessed using a structured questionnaire.

**Results:**

the prevalence of HBV in the LHD and MHD were 5.7% and 7.5% respectively. Pregnant women in the LHD demonstrated good knowledge but adopted poor practices whereas in the MHD, pregnant women demonstrated poor knowledge and adopted poor practices regarding the mode of transmission and prevention of HBV infection. There was a significant association between the prevalence of HBsAg and marital status (p = 0.000) in the LHD and age (p = 0.022) in the MHD.

**Conclusion:**

this study indicated a high prevalence of HBV among pregnant women in the LHD and MHD, knowledge and practices were identified as potential risk factors.

## Introduction

Hepatitis B infection is caused by the hepatitis B virus (HBV), an enveloped DNA virus that infects the human liver and causes hepatocellular necrosis and inflammation. It is transmitted through sexual intercourse with an infected person, by exchange of saliva during kissing with an infected person and also from infected mothers to their babies: during childbirth, breastfeeding and through the placenta [1]. HBV is a potentially life-threatening cause of liver diseases in the world. Liver injury occurs through immune-mediated killing of infected liver cells [2]. This infection can either be acute or chronic and may range from asymptomatic infection or mild disease to severe or rarely fulminant hepatitis. Acute hepatitis B infection is usually a self-limiting disease marked by acute inflammation and hepatocellular necrosis, with a case fatality rate of 0.5-1% [3]. Chronic hepatitis B infection encompasses a spectrum of disease and is defined as persistent HBV infection that is the presence of detectable hepatitis B surface antigen (HBsAg) in the blood or serum for longer than six months, with or without associated active viral replication and evidence of hepatocellular injury and inflammation [4]. Chronicity is common following acute infection in neonates and in young children under the age of 5 years, but occurs rarely when infection is acquired in adulthood [5]. Globally, WHO estimates that, more than 2 billion people are still living with HBV infection and over 350 million people are believed to be at risk of developing complications of chronic hepatitis such as cirrhosis and primary hepatocellular carcinoma [6, 7]. In the Global Burden of Disease 2010, the total number of deaths attributable to hepatitis B was 786,000 deaths, of which 132,200 (17%) were estimated to be caused by acute hepatitis B, 341,400 (43%) were caused by liver cancer and 312,400 (40%) were caused by cirrhosis [8].

In Africa and Asia, the prevalence of HBV is > 8% and 2 billion people have markers of current or past infection with HBV [9]. Approximately 65 million of all chronically infected individuals live in Africa [10]. In Cameroon, the prevalence of HBV ranges from 6-16% [11, 12]. Recent studies report the prevalence of HBV to be as high as 10.1% and 12% among blood donors in hospital blood banks in Cameroon [13, 14]. Frambo [11] reported a 9.7% prevalence of HBsAg among pregnant women in the Buea health district. HBV infection may run undetected. Delay in the diagnosis of HBV due to unawareness of the infection may lead to HBV related liver diseases [11]. Studies assessing knowledge and practices regarding HBV prevention and transmission is mostly conducted on health care workers since they are considered to be at a higher risk of acquiring HBV infection especially clinicians with direct patient contact as well as laboratory workers. However, pregnant women are also considered vulnerable and even at a higher risk of transmitting the virus to their newborns if not early diagnosed. Viral hepatitis during pregnancy is associated with high risk of maternal and infant complications leading to spontaneous abortions, premature delivery, intrauterine growth restrictions and low birth weight infants [11, 15, 16]. The risk of vertical transmission depends on the time at which a pregnant woman acquired HBV infection [17]. In the absence of immuno-prophylaxis, 10-20% of women seropositive for HBsAg transmit the virus to their neonates. It is for these reasons that this study was designed to determine the prevalence of HBsAg among pregnant women and to assess knowledge and practice of pregnant women attending ANC on HBV prevention and transmission.

## Methods

**Study design and population:** a cross-sectional retrospective review of hospital records and administration of questionnaires was carried out in Antenatal Clinics (ANC) in the Limbe and Muyuka Health Districts of the South West Region of Cameroon from July to August 2017. The ANCs involved in the study were those of the Regional Hospital Limbe, District Hospital Limbe, District Hospital Muyuka and Presbyterian Health Center Muyuka. These hospitals were selected on the basis that they have been screening pregnant women for Hepatitis B infection for the past 3years. Targeted subjects in this study were pregnant women attending ANC in all the hospitals under study who gave consent to take part in the study and review of hospital records of pregnant women who attended ANC from 2014-2016 to determine the prevalence of HBV infection.

### Selection criteria

**Inclusion criterion:** pregnant women who attended ANC during the period of data collection (July-August) and consented to participate in the study.

**Exclusion criterion:** pregnant women who did not consent to the study.

**Sample size and sampling:** the sample size for questionnaire administration was calculated using the Fisher's formula, with a prevalence of 9.7% of Hepatitis B infection [11] among pregnant women with an error margin (d) of 0.05 and a 95% confidence interval.

$$N=\frac{Z^{2}\;X\;P\;\left(1-\text{P}\right)}{d^{2}}$$

Therefore, N= (1.96² x 0.097 (1-0.097))/0.05² N=135. Therefore, sample size for questionnaire administration to pregnant women was 135 questionnaires per health district giving a total of 270 questionnaires. The sample size for review of hospital records was exhaustive. All complete records of women who attended ANC sessions in the study sites between 2014 and 2016 were considered. The sampling technique employed was an exhaustive sampling technique where pregnant women attending ANC available at the time of the study and who were willing to participate in the study were included for the purpose of the study.

**Data collection:** all participants who consented were interviewed using a structured questionnaire adapted from the questionnaire formulated by Mohammed [18]. Prior to its use in this study, a total of 12 questionnaires were pretested at the Regional Hospital Buea among pregnant women attending ANC with the aim of revising poorly structured questions and to estimate the average time required to fill the questionnaire. A total of 270 questionnaires were administered to pregnant women attending ANC in all the hospitals under study for a period of 2 months (July-August) to assess their knowledge and practices on Hepatitis B prevention and transmission. Knowledge on HBV infection consisted of 12 questions and each correct response was scored as 1 and 0 for a wrong response. The knowledge scores for an individual was calculated and summed up to give a total knowledge score on 12. A score between 0-4 was classified as poor, 5-8 as good and 9-12 as excellent adapted from a study conducted by Abongwa [16]. Practices of pregnant women on HBV infection were assessed on a scale of 6 since there were 6 questions on practices regarding HBV infection. A score of 0-3 was classified as poor practice while a score of 4-6 was classified as good practice. Demographic information of the participant was also obtained through administration of questionnaires.

**Data analyses:** data from questionnaires and hospital registers were entered into separate templates in Excel version 10. The data was verified for completion, cleaned and exported into SPSS v 16.0 for analyses. Descriptive analysis was carried out by calculating the mean, median, standard deviation and frequencies of different variables using SPSS v 16.0. The prevalence rates of hepatitis B virus infection were retrospectively calculated. Chi-square test was used to: assess if there is an association between prevalence of HBV infection and the different age of the participants; assess if there is an association between prevalence of HBV infection and parity of the participants; assess if there is an association between prevalence of HBV infection and marital status of the participants. Significant level was set at p < 0.05

**Ethical considerations:** ethical clearance was obtained from the Institutional Review Board of the Faculty of Health Science, University of Buea. Administrative clearance was obtained from the Regional Delegation of Public Health for South West Region Cameroon and written approval from the head of every hospital under study. Participants had the study protocol carefully explained to them and participation was voluntary. Written informed consent was obtained from all participants. Study participants, data confidentiality and integrity were maintained by restricting access of the information and primary data to the principal investigator.

## Results

**Socio-demographic characteristics of pregnant women who attended ANC in the Limbe and Muyuka Health Districts between 2014 and 2016:** the characteristics of the 2647 pregnant women who attended ANC in the Limbe and Muyuka Health Districts between 2014 and 2016 are summarized in Table 1. Their ages ranged from 14 to 47years with a mean ±SD age of 26.77 ± 5.713 years, with the predominant age group being 25-34years. About 1340 (50.6%) of the women were students and 13 (0.5%) practiced polygamous marriage, 982 (37.1%) of the women were at their first pregnancy (Table 1).

**Table 1:** demographic characteristics of pregnant women who attended ANC in the Limbe and Muyuka Health Districts between 2014 and 2016

Characteristics (n=270)	Stratification	Frequency	Percentage (%)
Age	<25	974	36.8
	25-34	1408	53.2
	˃35	265	10.0
	Total	2647	100
Occupation	civil servant	848	32.1
	Jobless	48	1.8
	Self employed	411	15.5
	Student	1340	50.6
	Total	2647	100
Area of residence	Urban	1360	51.4
	Rural	1287	48.6
	Total	2647	100
Marital status	Single	808	30.5
	Married monogamy	1779	67.2
	Married polygamy	13	0.5
	Co inhabitation	47	1.8
	Total	2647	100
Parity	First pregnancy	982	37.1
	Primipara(1)	654	24.7
	Multipara(2-4)	937	35.4
	Grand multipara(>4)	74	2.8
	Total	2647	100

**Prevalence of hepatitis B infection among pregnant women obtained by years and by Health Districts:** the prevalence of hepatitis B infection in pregnant women varied yearly according to the different health districts. It was observed that there were 12 (0.85%) cases in 2014, 32 (2.35%) cases in 2015 and 34 (2.5%) cases in 2016 in the Limbe health districts with p = 0.806. In the Muyuka health district, it was observed that there were 16 (1.21%) cases in 2014, 52 (4.04%) cases in 2015 and 29 (2.25%) cases in 2016 with p = 0.007. The highest number of cases was recorded in the year 2015.

**Figure 1:**
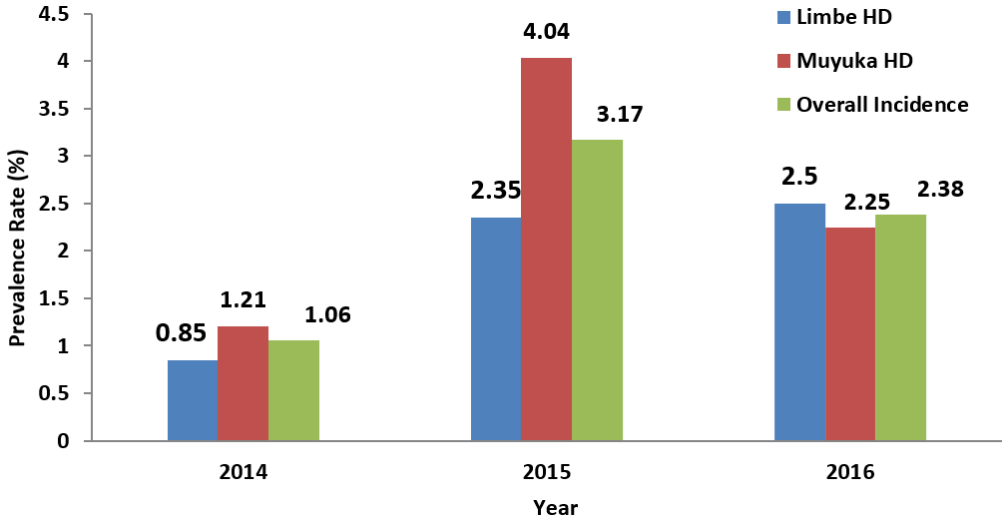
yearly prevalence of hepatitis B among pregnant women in the Limbe and Muyuka health districts

**Prevalence of hepatitis B in the Limbe and Muyuka Health Districts:** from 2014 to 2016, a total of 1360 and 1287 pregnant women were registered and tested for HBsAg in the Limbe and Muyuka health districts respectively. Out of these number 78 (5.7%) and 97 (7.5%) were positive for HBsAg in the Limbe and Muyuka health districts respectively. It was observed that there was a significant association between the prevalence of HBV and the health district (p=0.00) (Figure 1).

**Overall prevalence:** from 2014 to 2016, a total of 2647 were registered and tested for hepatitis B infection in the Limbe and Muyuka health districts. Of these number, 175 (6.6%) were positive for HBV infection.

**Relationship between prevalence of Hepatitis B infection and socio-demographic characteristics of pregnant women in the Limbe and Muyuka Health Districts:** it was observed that the prevalence of HBsAg varied according to age and marital status of the pregnant women in the Limbe and Muyuka health districts. However, the difference in prevalence of HBsAg was significant according to marital status (p = 0.000) and age (p = 0.022) in the Limbe and Muyuka health districts respectively, with those who practiced monogamous marriage having the highest prevalence (4.6%) in the Limbe health district (Table 2).

**Table 2:** relationship between age, marital status, parity and HBV infection in the Limbe and Muyuka Health Districts

		Limbe health District			Muyuka health district		
Characteristics	Level	Number tested (N)	Positive (%)	P value	Number tested (N)	Positive (%)	P value
Age	<24	401	1.54	0.719	573	3.19	0.022
	25-34	784	3.42		624	3.77	
	>34	175	0.74		90	0.54	
Marital status	Single	383	1.03	0.000	425	3.11	0.338
	Married monogamy	969	4.6		810	4.12	
	Married polygamy	6	0.0		7	0.07	
	Co inhabitation	2	0.07		45	0.2	
Parity	First Pregnancy	517	1.9	0.173	465	2.81	0.531
	Primipara (1)	283	1.1		371	2.27	
	Multipara (2-4)	532	2.4		405	2.35	
	Grand multipara (>4)	28	0.3		46	0.08	

**Socio-demographic characteristics of the 270 pregnant women who responded to questionnaires on hepatitis B infection in the Limbe and Muyuka Health Districts:** the ages of these women ranged from 16 to 46 years with a mean ± SD age of 22.6 ± 5.6 years, with the predominant age group being pregnant women of 25-34 years. About 78 (28.9%) of the pregnant women were students, 119 (44.1%) were polygamously married, 8 (3.0%) had multiple sexual partners and only 13 (4.8%) of them were muslims (Table 3).

**Table 3:** demographic characteristics of the 270 pregnant women attending antenatal clinic in the Limbe and Muyuka Health Districts, July-August 2017

Characteristics (n=270)	Stratification	Frequency	Percentage %
Age	<25	116	43.0
	25-34	130	48.1
	˃35	24	8.9
	Total	270	100
Occupation	civil servant	49	18.2
	Jobless	67	24.8
	Self employed	76	28.1
	Student	78	28.9
	Total	270	100
Area of residence	Urban	133	49.3
	Rural	137	50.7
	Total	270	100
Marital status	Single	121	44.8
	Married monogamy	119	44.1
	Married polygamy	22	8.1
	Divorced	3	1.1
	Widow	5	1.9
	Total	270	100
Parity	First pregnancy	107	39.6
	Primipara (1)	60	22.2
	Multipara (2–4)	102	37.8
	Grand multipara (>4)	1	0.4
	Total	270	100
Sexual Partner	Single	262	97.0
	Multiple	8	3.0
	Total	270	100
Religion	Christian	257	95.2
	Muslim	13	4.8
	Total	270	100

**Figure 2:**
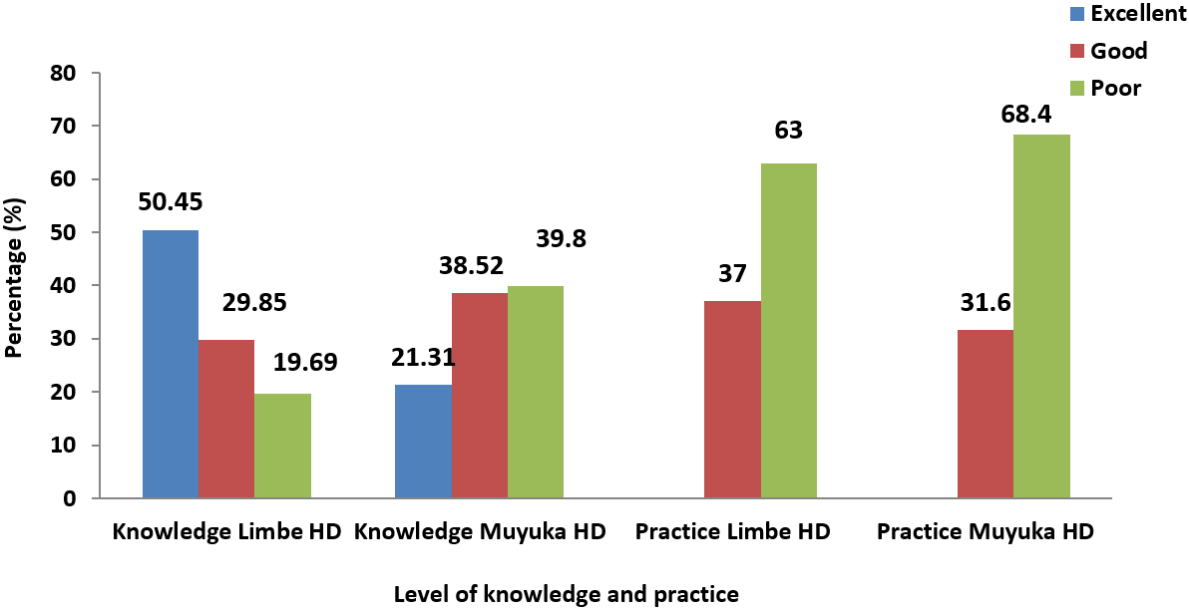
knowledge and practice of pregnant women in the Limbe and Muyuka health districts

**Scores on knowledge and practice of pregnant women on the transmission and prevention of Hepatitis B virus infection in the Limbe and Muyuka Health Districts:** out of the 135 pregnant women who responded to the questionnaires in the Limbe health district, 68 (50.45%) demonstrated excellent knowledge, 40 (29.85%) had good knowledge and 27 (19.69%) had poor knowledge on the transmission and prevention of hepatitis B virus infection. Regarding practices of pregnant women on transmission and prevention of hepatitis B, 50 (37%) of them were classified as those adopting good practices in the transmission and prevention of hepatitis B virus infection and 85 (63%) as those adopting poor practices. Of the 135 women who responded to the questionnaires in the Muyuka health district, 29 (21.31%) demonstrated excellent knowledge, 52 (38.52%) had good knowledge and 54 (39.8%) had poor knowledge on the transmission and prevention of Hepatitis B virus infection. Regarding practices of pregnant women on transmission and prevention of hepatitis B, 43 (31.6%) of women were classified as adopting good practices and 92 (68.4%) as having poor practices (Figure 2).

## Discussion

The prevalence of HBV infection was estimated among pregnant women based on records obtained, knowledge and practices regarding hepatitis B prevention and transmission of pregnant women attending ANC in the Limbe and Muyuka health districts of the South West region of Cameroon were assessed. The data gathered in this work may serve to add information to influence policy on the prevention and control of hepatitis B infection in Cameroon. The prevalence rate of hepatitis B among pregnant women attending antenatal clinic in the Limbe health district was 5.7%. This prevalence rate is similar to the 5.44% prevalence of HBsAg in pregnant Lao women [19] and 5.9% in the Democratic Republic of Congo [20]. However, the prevalence rate in this study is lower than the 9.7% in Buea health district [11] and 7.7% in the North West region of Cameroon [21]. The prevalence rate in this study is higher than the 1.94% seroprevalence of HBsAg among pregnant women attending ANC clinic in Ethopia [22]. The observed difference in HBV distribution across the different geographical location might be attributed to variation in socio demographic characteristics of the study population such as socio-cultural environment, cultural practices, sexual practices, medical exposure and the difference in hepatitis epidemiology. The variations might also be due to methodological differences, the level of awareness, cultural and behavioral differences for the potential risk factors of HBV infection as indicated by Esan [15].

In Muyuka, a prevalence rate of 7.5% of HBsAg among pregnant women was recorded. This was higher than the 5.7% prevalence of HBsAg in the Limbe health district. This can be explained by the fact that women in the Limbe health district considered as an urban area, are more knowledgeable on the mode of prevention and transmission of hepatitis B virus infection as compared to women in the Muyuka health district. The 7.5% prevalence of HBsAg among pregnant women in the Muyuka health district is an indication that pregnant women serve as an important reservoir to fuel the HBV infection in the general population. The prevalence of 7.5% of HBsAg in the Muyuka health district is however, similar to the 8.3% prevalence of HBsAg in rural settings in Nigeria [23] and 7.9% in Yaounde [24], although lower than the 12.14% in Yaounde [13] and 20.4% in a semi-urban area in the North region of Cameroon [25] in a study constituting both male and female. This shows that prevalence of HBV varies in different regions and in different groups of the same population. These differences in different countries might be due to variability in ethnicity, high rate of emigration due to urbanization, geographical regions, genetic factors and socioeconomic status associated with peculiarities in the modes of transmission and cultural practices [19]. The highest prevalence of HBsAg was detected in pregnant women who were on their first pregnancy. This is in contrary to the results of Ndams [26] in Minna, Nigeria. The difference in the prevalence of HBsAg among the age groups was statistically significant (p = 0.022) in the Muyuka health district. This contradicts the observation by Abongwa and Kenneth [21] in their study on hepatitis B among pregnant women in Bamenda where there was no significant difference between the age of participants and the prevalence of HBsAg (p = 0.29). Our observation in the Limbe health district on the prevalence of HBsAg among the age groups (p = 0.233) was however similar to that of Abongwa and Kenneth [21] where there was no difference in the prevalence (p = 0.29) of hepatitis B infection among the age groups in the North West region of Cameroon. There was also a significant difference between marital status and the prevalence of HBsAg (p = 0.000) in the Limbe health district. HBV prevalence was however high among women between the age group 25-34 years (3.1%). This age group is sexually very active, suggesting the role of sexual intercourse in the transmission of HBV. However, it was contrary to studies carried out by [23, 27] who stated that the prevalence was high among women in the age group 21-25 years.

Results of our study, shows that pregnant women in the Limbe health district demonstrated a good to excellent knowledge but adopted poor practices regarding the mode of transmission and prevention of hepatitis B virus infection. Good knowledge of pregnant women regarding the mode of transmission and prevention of hepatitis B virus infection seen in this study is similar to studies reported in Egypt [28] and in Japan [29]. On the contrary, studies from Egypt showed good practices regarding hepatitis B [28]. Good knowledge of pregnant women in the Limbe health district regarding the mode of transmission and prevention of hepatitis B virus infection can be explained by the fact that these women have been receiving regular ANC talks on the subject of hepatitis B infection. On the contrary, in the Muyuka health district, pregnant women demonstrated poor knowledge and adopted poor practices regarding the mode of transmission and prevention of hepatitis B virus infection. This is similar to findings in the North West region of Cameroon and Buea health district, where pregnant women demonstrated poor knowledge and adopted poor practices regarding the mode of transmission and prevention of hepatitis B virus infection [11, 16]. Our observation is also similar to those reported in Pakistan [7, 30]. The poor knowledge of pregnant women regarding the transmission and prevention of HBV in the Muyuka health district can be explained by the lack of formal education available about HBV as compared to other diseases of similar modes of transmission and burden among the pregnant women, many of whom come from the rural villages. This poor knowledge warrants the need for sustained and continuous education about hepatitis B virus infection.

**Some limitations in our study include:** limited demographic information available for pregnant women; the fact that we did not test the pregnant women for HBsAg and or HBeAg but rather collected data from hospital records to determine the prevalence of HBsAg. Furthermore, studying self-reported knowledge and practices is itself a limitation as one cannot rely totally on the information provided by the participants because of recall bias and social desirability bias. Despite these shortcomings, this study provides relevant information in the context of very limited epidemiological data on HBV infection in the South West region of Cameroon, especially among pregnant women in the semi-urban milieu.

## Conclusion

This study investigated the prevalence of HBsAg and assessed knowledge and practice of pregnant women regarding HBV prevention and transmission in the Limbe and Muyuka health districts. From the results obtained, we conclude as follows: the prevalence of HBsAg among pregnant women is 5.7% and 7.5% in the Limbe and Muyuka health districts respectively; knowledge on hepatitis B infection constituted a risk factor in the Muyuka health district; practices regarding hepatitis B infection and transmission constituted risk a factor in both the Limbe and Muyuka health districts.

### What is known about this topic

- In the far north region of Cameroon, the prevalence of HBV infection was reported to be 10.2% among pregnant women with 1.5% of these women coinfected with HIV;
- The prevalence of HBV is >8% in Africa and Asia and 2 billion people have markers of current or past infection with HBV;
- In sub-Saharan Africa, exposure to HBV remains a serious risk to health care workers. It has been estimated that 6200 HBV infections occur each year among health care workers in sub-Saharan African.

### What this study adds

- About 3.42% and 3.77% of pregnant women found positive for HBsAg were between the age 25-34 years in the Limbe and Muyuka health districts respectively;
- In the Limbe health district, 37% of pregnant women had good practices regarding HBV prevention and transmission meanwhile 31.6% had good practices regarding HBV prevention and transmission in the Muyuka health district;
- In the Limbe and Muyuka health districts, the prevalence of HBsAg was 5.7% and 7.5% respectively.

## Competing interests

The authors declare no competing interests.
